# Compliance to recall visits by patients with periodontitis — Is the practitioner responsible?

**DOI:** 10.4103/0972-124X.70829

**Published:** 2010

**Authors:** Angel Fenol, Simi Mathew

**Affiliations:** *Department of Periodontics, Amrita School of Dentistry, Amrita Institute of Medical Sciences, Ponekkara PO, Kochi - 682 041, Kerala, India*; 1 *Department of Community Dentistry, Amrita School of Dentistry, Amrita Institute of Medical Sciences, Ponekkara PO, Kochi - 682 041, Kerala, India*

**Keywords:** Compliance, recall visits, supportive periodontal therapy

## Abstract

**Context:**

Compliance to recall visit is directly related to the medium and long-term success of active periodontal therapy.

**Aims:**

To determine the percentage of patients who were compliant to recall visits by the practitioner and to find out the reasons for noncompliance by noncompliant patients.

**Settings and Design:**

Cross-sectional study was carried out in the Department of Periodontology, Amrita School of Dentistry. The study participants were former patients of the Department of Periodontology.

**Patients and Methods:**

A total of 216 patients were selected for the study — 116 males and 100 females. They were divided according to their socioeconomic status — professionals and nonprofessionals. Nonprofessionals were divided into those who had completed high school education and those who had not. They were followed up to find out how many were compliant to recall visits and the reasons for noncompliance by noncompliant patients.

**Statistical Analysis:**

The data was entered into SPSS version 11.5. Descriptive statistics were used. The frequencies of responses were calculated.

**Result:**

Of the total study population, 48.1% was compliant, of which 58.6% and 37.6% of males and females were compliant, respectively. In terms of percentage, 60.6% of professionals, 52.4% of those who had completed high school education and 31.3% of those who had not completed high school education were compliant.

**Conclusion:**

Compliance to recall visits by the periodontitis patients depends largely on the practitioner. Inadequate motivation by the practitioner and inadequate education in general are responsible for noncompliance to periodontal treatment.

## INTRODUCTION

Supportive periodontal therapy (SPT) is directly related to the medium and long-term success of active periodontal therapy.[1–3] Unlike other diseases of the oral cavity that produce acute pain and discomfort, patients with periodontal disease do not feel the need to be compliant with the recall visits scheduled by the practitioner. According to Robert L. Merrin, prevention of periodontal disease requires as positive a program as that required for the elimination of periodontal diseases. In previous studies by Novaes *et al*., the results showed that 26% of the treated patients returned for SPT; and of those, 40% returned irregularly. This paper discusses on the basis of a study conducted to ascertain how compliant the patients were after initial treatment in the department. This highlights the importance of the effort that can be put in by the practitioner so that the percentage of patients who are compliant to recall visits can be increased.

### Objective the study

To determine the percentage of patients compliant to further treatment and recall visits after the initial therapy and to find out the reasons for the same.

## PATIENTS AND METHODS

A total of 216 patients were taken up for the study. Of these, 116 were males and 100 were females. They had either their consultation done or their etiotropic phase completed. They were also divided according to their socioeconomic status — professionals and nonprofessionals. Nonprofessionals were divided into those who had completed high school education and those who had not. There were 66 professionals and 150 nonprofessionals. Of the nonprofessionals, 112 had not completed high school education and 38 had completed high school education. They were followed up to find out how many were compliant to recall visits and the reasons for noncompliance by noncompliant patients.

A telephonic interview was done using the 5-item structured questionnaire including the following aspects:

1. Good experience
2. Bad experience
3. Inadequate information about the disease and treatment options
4. Inadequate motivation by the practitioner
5. Forgot the appointment

### Statistical analysis

The data was entered into SPSS version 11.5. Descriptive statistics were used. The frequencies of responses were calculated.

## RESULTS

**Figure 1:**
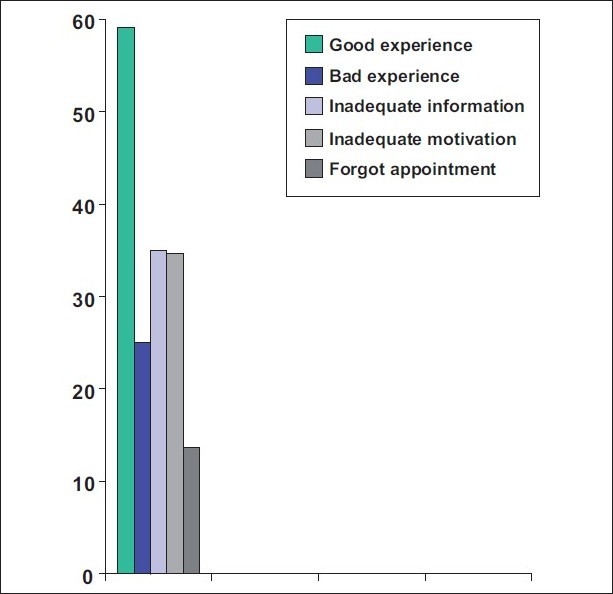
Percentage of patients with good or bad experience, those with inadequate information and motivation and those who forgot their appointments

Figure 1 shows Good experience — 59.1%; Bad experience — 25%; Inadequate information about the disease and treatment options — 35%; Inadequate motivation by the practitioner — 34.6%; Forgot the appointment — 13.65%

**Figure 2:**
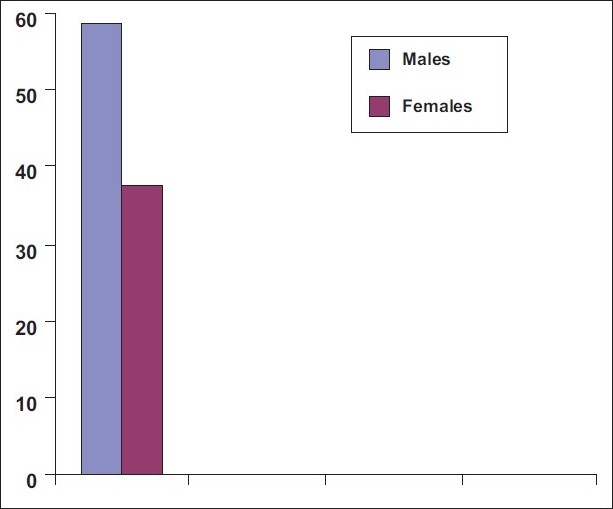
58.6% of males 37.6% of females were compliant

Figure 2 shows 48.1% of the total study population was compliant. According to gender, 58.6% of males and 37.6% of females were compliant.

**Figure 3:**
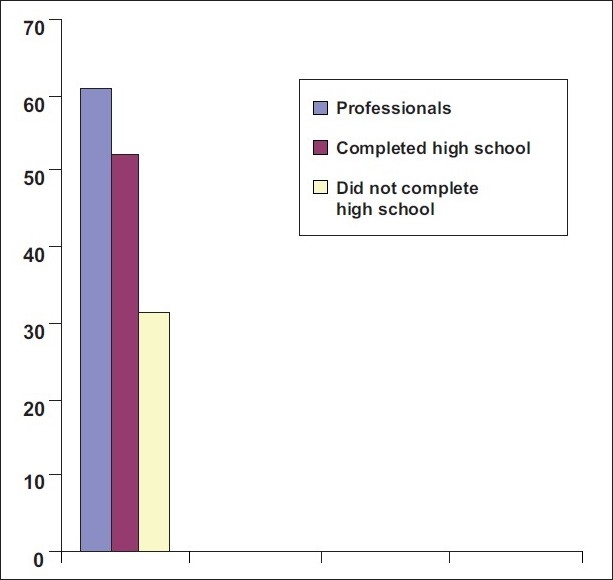
60.6% of professionals, 52.4% of those who completed high school, 31.3% of those who did not complete high school were complaint

Figure 3 shows 60.6% of professionals, 52.4% of those who had completed high school education and 31.3% of those who had not completed high school education were compliant.

## DISCUSSION

Adherence to the schedule for recall dental appointments after treatment is believed to be the key in preventing recurrence of periodontal disease.[4] In this study, 48.1% of the patients were compliant with recall visits to the dentist. In terms of percentage, 34.6% of the study patients informed that they were inadequately motivated by the practitioner.[5] This was similar to the study done by Novaes *et al*., who said that 40% returned irregularly. To achieve this goal of making the patients more compliant, the clinician must be open and honest with the clients regarding the oral conditions and the new techniques currently available. It must be kept in mind that unrealistic treatment options must be avoided. The dental professional has to spend time to discuss the plan in detail. For this, he/she requires to give an effective case presentation, which requires excellent communication skills, which is necessary for client motivation.[6] In order to achieve a concise presentation, practitioners need to have their thoughts organized well in advance of their presentation. Visual aids such as those related to periodontal status assessment, radiographs and illustrations will be helpful.

Inadequate information about the disease in terms of what would happen if the disease was left untreated and the treatment options available was reported by 35% of the patients. When clients are given the opportunity to understand their disease and the remedies that are available today, acceptance of periodontal therapy recommendation becomes a near certainty. A similar study done by Novaes *et al*. showed that 26% of the treated patients returned for SPT; and of those, 40% returned irregularly.[7] In a Brazilian study, in the surgical group, 43.9% were noncompliant; and in the nonsurgical group, 53.2% were noncompliant.[8]

In this study, 13.65% failed to keep their appointments. Failed appointments create problems for both the patient and the therapist. It is necessary to use appropriate vehicles for reminders, including e-mails, postcards and telephone calls, about the appointment well in advance and even on the day of the appointment.

It was also seen that more males than females were compliant to recall visits (58.6 % and 37.6%, respectively). The professionals were more compliant (60.6%). This reflects that they understood the importance of recall visits and the explanation given by the practitioner. The people in the lower socioeconomic group were found to be less compliant (31.3%). This, again, highlights the importance of the need to strengthen the efforts put in by the practitioner to make the patients in this group more compliant.

The strength of this study is that it was done in a hospital setup where patients from all socioeconomic status come for treatment. This is the first study of its kind done in our setup to understand why patients were not being compliant to periodontal therapy. The study is a cross-sectional, descriptive study. A longitudinal study would provide a more accurate estimation of the compliance by the patients.

At present a systematic review is not available, and not much work has been done in the context of compliance to recall visits to the periodontist.

According to the findings from this study, practitioners need to put in more efforts towards improving patient care. They need to improve their communication skills, which would require them to attend continuing education programs relating to the same. They should seem genuinely interested in the welfare of their patients. All treatment options and monetary affairs should be adequately explained to the patients, and the patients can be reminded of their appointments in advance.

## CONCLUSION

Frequent reevaluation and careful monitoring provide practitioners with the opportunity to intervene early in the disease state, to reverse or arrest the progression of periodontal disease with meticulous nonsurgical anti-infective therapy.

Compliance to recall visits by the patients is the responsibility of both the practitioners and the patients. Adequate steps should be taken to ensure that the patients understand their disease, the treatment options available and the consequences if appropriate treatment is not done. All this requires good communication skills, and the practitioners should be honest and the patients should feel that the practitioners are genuinely interested in their care. Patients should also be reminded about their appointments well in advance so that their appointments are not missed. All these points if kept in mind and adequately practiced will drastically increase the compliance by the patients to recall visits to the practitioner.
